# Integrated Network Pharmacology and Single-Cell Transcriptomics Reveal Transketolase as a Potential Target for the DanShen–DaHuang Herb Pair in Acute Kidney Injury

**DOI:** 10.3390/ijms27104435

**Published:** 2026-05-15

**Authors:** Yang Zhang, Haolan Yang, Jin Li, Xinyan Wu, Lixia Li, Gang Ye, Kun Zhang, Zhijun Zhong

**Affiliations:** 1 College of Veterinary Medicine, Sichuan Agricultural University, Chengdu 611130, China; 2022103024@stu.sicau.edu.cn (Y.Z.); yanghaolan1@stu.sicau.edu.cn (H.Y.); 2025303136@stu.sicau.edu.cn (J.L.); lixiali@sicau.edu.cn (L.L.); 13521@sicau.edu.cn (G.Y.); 72011@sicau.edu.com (K.Z.); 2 College of Food Science and Nutritional Engineering, China Agricultural University, Beijing 100000, China; xinyan_wu@cau.edu.cn

**Keywords:** AKI, DanShen–DaHuang, transketolase, network pharmacology, single-cell transcriptomics

## Abstract

Acute kidney injury (AKI) lacks targeted pharmacological interventions. While the DanShen–DaHuang (DS-DH) herb pair shows clinical potential for AKI treatment, and our prior study has validated its nephroprotective efficacy in a cisplatin-induced murine model, its specific molecular targets within the renal microenvironment remain undefined. In this study, we integrated network pharmacology and weighted gene co-expression network analysis (WGCNA) to screen AKI-related targets of the DS-DH pair. A multi-algorithmic machine learning pipeline (including LASSO, Boruta, Random Forest, GBM, XGBoost, and Decision Trees) was utilized to calculate feature importance scores and rank core genes. Subsequently, single-cell RNA sequencing (scRNA-seq) data (GSE197266) were analyzed for transcriptomic mapping, pseudotime trajectory, and cell–cell communication. Finally, molecular docking evaluated theoretical binding affinities. After database screening, a total of 603 drug–disease intersecting targets were obtained. Subsequently, 917 module genes significantly associated with AKI were identified by WGCNA, and 62 core candidate genes were determined after intersecting with the above targets. Multi-algorithm machine learning ranked the importance of the 62 targets, with transketolase (TKT) ranking the highest. To elucidate the mechanism of TKT in AKI, scRNA-seq analysis was performed on 77,593 high-quality cells. The results showed that *Tkt* was specifically enriched in renal macrophages, with the highest expression in the M2-polarized subset. Pseudotime analysis further revealed that *Tkt* expression dynamics were highly synchronized with the differentiation trajectory of M2 macrophages and positively correlated with the repair markers *Arg1* and *Mrc1*. Cell–cell communication analysis predicted that *Tkt*^+^ M2 macrophages act as active communication hubs via the *Spp1* and *Mif* signaling axes. Molecular docking validated the favorable binding affinity between core DS-DH compounds and the TKT active pocket. This computational framework predicts that the DS-DH herb pair might mitigate AKI by potentially targeting TKT, a metabolic enzyme closely associated with macrophage M2 polarization. By prioritizing targets via multi-algorithmic scoring, we provide a data-driven rationale and candidate targets for future experimental validation.

## 1. Introduction

Acute kidney injury (AKI) is a severe and highly prevalent clinical syndrome characterized by a rapid, often irreversible decline in renal excretory function. Globally, AKI affects millions of hospitalized individuals annually, contributing significantly to an unacceptably high mortality rate and an enormous socio-economic burden [1,2,3]. The pathophysiology underlying AKI is highly complex and multifactorial, involving a detrimental cascade of tubular epithelial cell necrosis, severe microvascular dysfunction, oxidative stress, and robust innate immune responses [4,5,6]. During the acute phase, the massive infiltration of inflammatory cells and the subsequent release of pro-inflammatory cytokines exacerbate tissue damage, which can ultimately drive the progression toward chronic kidney disease (CKD) if left unresolved. Among these infiltrating immune cells, macrophages play a particularly decisive role, as their phenotypic switch from a pro-inflammatory (M1) to a pro-reparative (M2) state is a critical determinant of whether the kidney undergoes successful repair or progresses to fibrosis [7]. Therefore, elucidating the molecular drivers of macrophage polarization within the complex renal milieu is essential for identifying novel therapeutic targets that can promote endogenous repair. Despite considerable advancements in renal replacement therapies and supportive care, effective targeted pharmacological interventions remain critically limited [8,9,10]. This therapeutic bottleneck is primarily due to the incomplete understanding of the intricate molecular mechanisms and regulatory networks that govern the critical transition from acute tissue injury to functional structural repair, highlighting an urgent unmet clinical need for novel therapeutic strategies [11,12].

Traditional Chinese Medicine (TCM) has garnered increasing attention as a complementary approach for kidney diseases due to its multi-component, multi-target characteristics. Recent studies have demonstrated that various herbal formulations and their bioactive constituents exhibit nephroprotective effects through anti-inflammatory, antioxidant, and anti-fibrotic mechanisms [13,14,15]. These findings underscore the potential of TCM as a source for developing novel renoprotective strategies.

Among these formulations, the DanShen–DaHuang (DS-DH) herb pair is a clinically utilized combination that has shown particular promise for renal disorders. Salvia miltiorrhiza (DanShen) is a medicinal herb that has been reported to improve microcirculation and inhibit platelet aggregation [16], and its extracts exert anti-inflammatory, antioxidant, and anti-apoptotic effects [17,18]. Preclinical and clinical studies have investigated its potential benefits in inflammatory and ischemic conditions, such as myocardial infarction, cerebral ischemia, and hepatic injury, although the level of clinical evidence varies across indications [19,20]. Specifically within the context of AKI, DanShen and its primary bioactive constituents (such as salvianolic acids and tanshinones) have shown significant efficacy in mitigating tubular apoptosis, improving renal microcirculation, and suppressing oxidative stress cascades [21,22].

Similarly, DaHuang (Rheum palmatum) is a frequently used botanical drug in TCM known for its strong purgative, detoxifying, and anti-inflammatory properties. Its active anthraquinone derivatives, notably emodin and rhein, have been systematically reviewed for their pharmacological activities and molecular mechanisms [23,24]. In renal pathologies, DaHuang actively attenuates systemic inflammation, reduces uremic toxin accumulation, and significantly inhibits the progression of renal interstitial fibrosis by modulating key pro-fibrotic signaling pathways [25,26]. Based on their complementary pharmacological profiles, the DanShen–DaHuang (DS-DH) herb pair is frequently employed as a classic, rationally designed combination in clinical practice. The integration of DanShen’s microvascular-protective properties with DaHuang’s potent anti-inflammatory and detoxifying capacities presents a promising clinical prospect, yielding reported synergistic effects in the comprehensive management of renal failure compared to single-herb treatments [27,28].

However, despite the extensive clinical utility and empirical success of the DS-DH pair, the precise molecular mechanisms underlying their combined intervention in AKI treatment remain largely undefined. Historically, the majority of existing pharmacological research has predominantly focused on isolating the effects of individual herbs or single active monomers [27]. This reductionist approach leaves the synergistic, multi-target network interactions of the combined botanical formulation entirely unexplored. Since the therapeutic superiority of TCM fundamentally relies on the synergistic interactions among multiple bioactive compounds modulating interconnected biological pathways simultaneously, the lack of a comprehensive, systems-level understanding severely limits the targeted clinical optimization and scientific interpretation of the DS-DH pair in modern medicine [29].

Deciphering the “multi-component, multi-target, multi-pathway” nature of this classic herb pair inherently requires advanced, high-dimensional analytical frameworks. Network pharmacology has emerged as a robust systems biology paradigm to systematically decode the complex mechanisms of botanical drugs [30]. By constructing and analyzing drug–target–disease interaction networks, this computational approach allows for the systematic prediction and prioritization of key therapeutic hubs. Furthermore, the recent advent of single-cell RNA sequencing (scRNA-seq) provides unprecedented transcriptomic resolution, enabling researchers to map dynamic gene expression changes across highly specific, heterogeneous cell populations [31]. Traditional bulk-tissue sequencing often masks the nuanced cellular crosstalk and rare cell dynamics within the complicated renal microenvironment. By contrast, scRNA-seq overcomes these critical limitations, offering a detailed spatiotemporal landscape of the intricate cellular responses during the injury and resolution phases of AKI.

However, despite the extensive clinical utility and empirical success of the DS-DH pair, the precise molecular mechanisms underlying their combined intervention in AKI treatment remain largely undefined. Our group has recently demonstrated in a cisplatin-induced murine model that the DS-DH combination effectively attenuates AKI [32]. Specifically, integrated multi-omics analysis revealed that DS-DH modulated the gut–kidney axis and broadly inhibited the renal MAPK signaling pathway, thereby reducing inflammation. While these findings provided a macro-level pharmacological landscape, they did not resolve which specific cell types and molecular targets within the heterogeneous renal microenvironment are directly engaged by DS-DH components. The MAPK pathway is a ubiquitous signaling cascade expressed across multiple renal cell populations; inhibiting it globally does not pinpoint the primary responder cells or the direct binding targets responsible for therapeutic synergy.

To address this gap without a priori bias toward any single mechanism, the present study employs a systematic computational pipeline integrating network pharmacology, multi-algorithm machine learning, and single-cell transcriptomics. The significance of this study is to advance from a drug-pathway understanding to a drug–target–cell paradigm, spatiotemporally mapping the “multi-component, multi-target” interaction of an herbal pair onto the dynamic renal cellular landscape. Crucially, this in silico strategy is designed as a hypothesis-generating engine, systematically predicting and ranking candidate targets to guide subsequent experimental validation toward the most promising cell-specific nodes.

## 2. Result

### 2.1. Identification of Core Targets for the DS-DH Herb Pair in AKI via Integrated Network Pharmacology and WGCNA

To investigate the molecular basis of DS-DH intervention in AKI, we integrated network pharmacology with WGCNA after retrieving the active constituents of DanShen and DaHuang from the TCMSP database.

**Figure 1:**
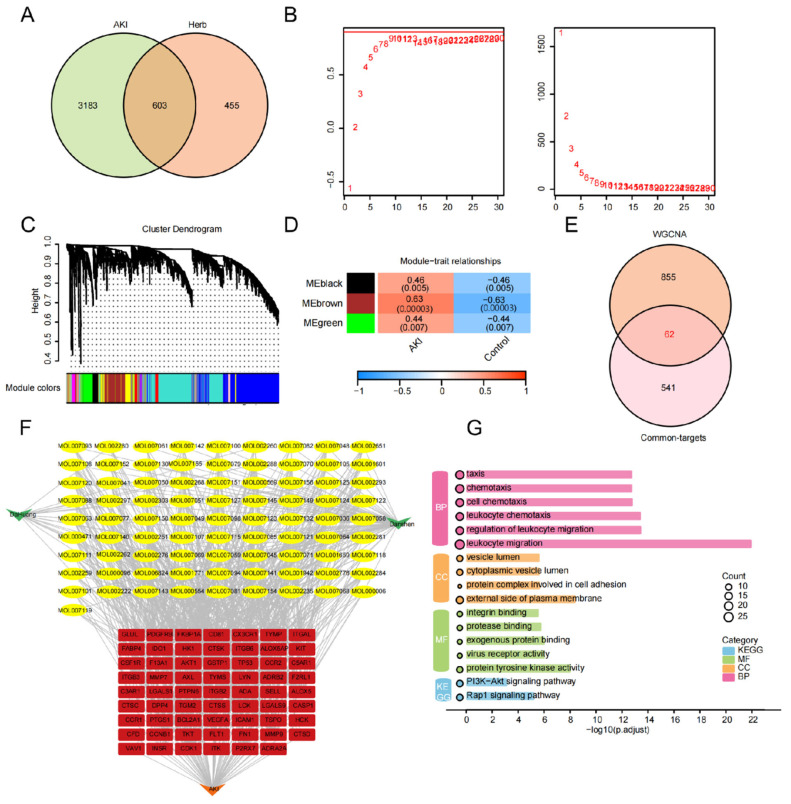
Identification and functional enrichment analysis of core targets for DS-DH in AKI. (**A**) Venn diagram showing the intersection of 1058 drug targets (from SwissTargetPrediction) and 3786 AKI-related genes (from GeneCards), resulting in 603 candidate targets. (**B**) Analysis of network topology for various soft-thresholding powers. The left panel shows the scale-free fit index, and the right panel shows the mean connectivity as a function of soft-thresholding power. (**C**) Hierarchical clustering dendrogram of genes identified by WGCNA. Each branch represents a gene module, with different colors denoting distinct module assignments. (**D**) Heatmap of the correlation between module eigengenes (MEs) and clinical traits (AKI vs. Control). The MEbrown (r = 0.63, *p* < 0.001), MEblack (r = 0.46, *p* = 0.005), and MEgreen (r = 0.44, *p* = 0.007) modules show significant positive correlations with AKI. (**E**) Venn diagram showing the 62 core targets identified by intersecting WGCNA key module genes (917) with candidate drug targets (603). (**F**) Component–Target–Disease interaction network of DS-DH in AKI. Red nodes represent core targets, including TKT and AKT1; yellow nodes represent active compounds; the green node represents the disease (AKI). Edges indicate compound–target interactions. (**G**) GO biological process and KEGG pathway enrichment analysis of the 62 core targets. The bubble plot highlights significantly enriched pathways related to leukocyte migration, chemotaxis, and PI3K-Akt signaling. Bubble size corresponds to gene count, and color intensity reflects the adjusted *p*-value.

Applying filters of OB ≥ 30% and DL ≥ 0.18, we identified 65 active compounds from DanShen and 17 from DaHuang (Supplementary Table S1). Target prediction for these 82 compounds via the SwissTargetPrediction database yielded 1058 potential pharmacological targets (Supplementary Table S2). Concurrently, 3786 AKI-related genes were sourced from the GeneCards database. The intersection of drug targets and disease-associated genes yielded 603 candidate targets for the DS-DH pair in AKI (Figure 1A). To refine the core regulatory network, WGCNA was performed on transcriptomic data. Upon constructing a scale-free network (Figure 1B,C), three core modules significantly correlated with the AKI pathological state were identified: MEbrown (r = 0.63, *p* < 0.001), MEblack (r = 0.46, *p* = 0.005), and MEgreen (r = 0.44, *p* = 0.007) (Figure 1D). Here, “ME” denotes module eigengene, and the color labels refer to arbitrarily assigned module identifiers in the WGCNA framework. By intersecting the union of these 917 module genes with the 603 drug–disease targets, we ultimately narrowed the focus to 62 core candidate targets (Figure 1E). The “Herb–Component–Target” interaction network (Figure 1F) highlights a synergistic multi-component regulatory pattern, with TKT, AKT1, MMP9, and CD44 emerging as central nodes. GO and KEGG enrichment analyses (Figure 1G) further revealed that these core targets are predominantly involved in leukocyte migration, chemotaxis, and the PI3K-Akt signaling pathway.

### 2.2. Deep Screening of Core Feature Genes Based on Multi-Algorithmic Machine Learning

**Figure 2:**
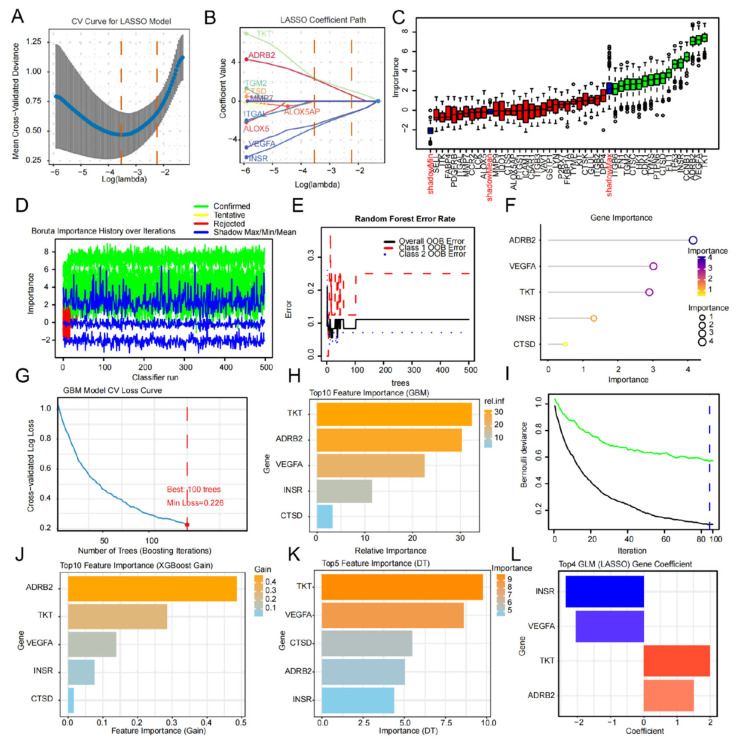
Identification of hub diagnostic genes in AKI using a multi-machine learning integration strategy. (**A**,**B**) LASSO regression analysis. (**A**) Cross-validation curve for optimal penalty parameter (lambda) selection; the dotted vertical line indicates lambda.min. (**B**) LASSO coefficient profile plot showing the shrinkage of feature coefficients with increasing lambda. (**C**,**D**) Boruta algorithm results. (**C**) Importance history of confirmed (green), tentative (yellow), and rejected (red) features across 500 iterations. (**D**) Final decision plot showing the Z-score importance of each feature relative to shadow features. (**E**,**F**) Random Forest model. (**E**) Relationship between out-of-bag error rate and the number of trees. (**F**) Ranking of gene importance based on Mean Decrease Gini. (**G**–**I**) Gradient Boosting Machine (GBM) model. (**G**) Cross-validation loss curve showing model convergence. (**H**) Top 10 feature importance ranking. (**I**) Bernoulli deviance plot. (**J**) Feature importance ranking based on XGBoost Gain score. (**K**) Feature importance ranking from the Decision Tree (DT) model. (**L**) Contribution coefficients of the top 4 core genes (TKT, ADRB2, VEGFA, and MMP9) derived from the Generalized Linear Model (GLM). TKT ranked consistently among the top contributors across all models.

As illustrated in Figure 2, a hierarchical screening strategy utilizing multiple machine learning algorithms was employed to identify the primary drivers of AKI progression from the 62 candidate targets. We first applied LASSO regression with cross-validation to determine the optimal penalty parameter, effectively eliminating collinearity while compressing the feature space (Figure 2A,B). Simultaneously, the Boruta algorithm was implemented, utilizing 500 iterations to compare the importance of real features against shadow features, thereby ensuring the robustness of our selection (Figure 2C,D). The intersection of results from LASSO and Boruta defined our consolidated candidate feature gene set (Supplementary Table S3). To evaluate the predictive contribution of these genes, ensemble learning algorithms were introduced. In the Random Forest model, gene importance was assessed by minimizing out-of-bag error (Figure 2E,F). Concurrently, gradient boosting machines (GBM) (Figure 2G–I), XGBoost (Figure 2J), and Decision Trees (DT) (Figure 2K) were utilized for multi-dimensional importance ranking. Finally, contribution coefficients for core predictive genes were determined using Generalized Linear Model (GLM) fitting (Figure 2L). The cross-algorithm analysis demonstrated that TKT, ADRB2, and VEGFA exhibited exceptionally high feature contributions and consistency across all models. Notably, TKT ranked among the top contributors in several non-linear models such as GBM and DT, suggesting its pivotal role as a transcriptional switch or metabolic hub in the DS-DH treatment of AKI.

### 2.3. Construction of a Single-Cell Atlas for AKI

**Figure 3:**
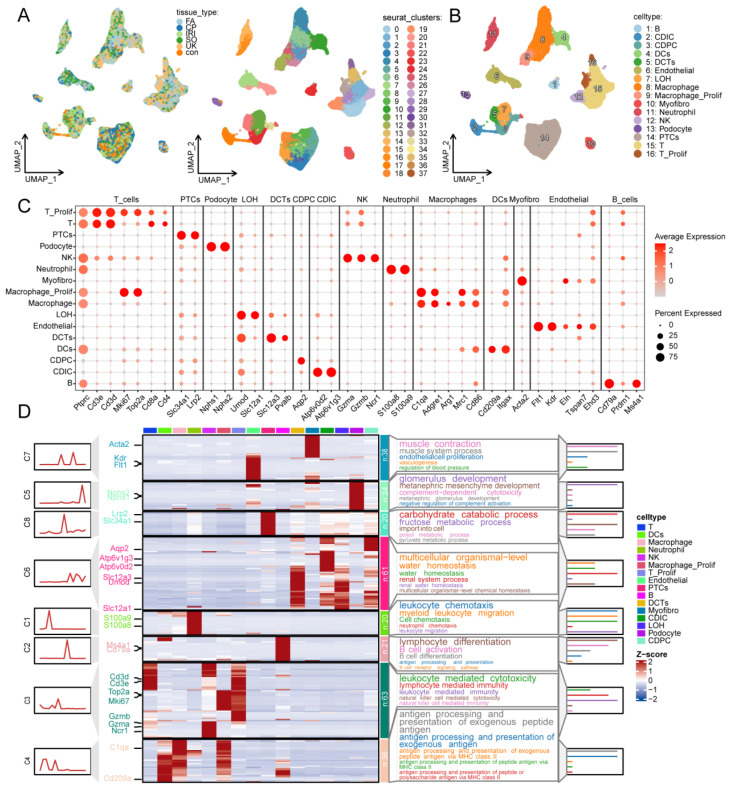
Single-cell transcriptomic landscape and functional characterization of renal cells in AKI. (**A**) UMAP visualization of renal cells. Left panel: cells colored by tissue origin (Control, IRI, CP, FA, SO, UUO). Right panel: cells colored by Seurat clusters. Abbreviations: IRI, ischemia–reperfusion injury; CP, cisplatin; FA, folic acid; SO, sodium oxalate; UUO, unilateral ureteral obstruction. (**B**) Cell type annotation based on canonical marker expression. Sixteen major cell types were identified, including podocytes, proximal tubule (PT) cells, macrophages, T cells, NK cells, endothelial cells, and collecting duct cells. (**C**) Dot plot of lineage-specific marker genes. Dot size represents the percentage of cells expressing the marker, and color intensity indicates the average expression level. Markers used for annotation: *Nphs1/Nphs2* (podocytes), *Slc34a1/Lrp2* (PT cells), *C1qa/Adgre1* (macrophages), *Ptprc/Cd3d* (T cells), *Gzma/Ncr1* (NK cells), and *Flt1/Kdr* (endothelial cells). (**D**) Hierarchical clustering heatmap (left) and GO biological process enrichment (right) of cell-type-specific signature genes. The heatmap displays Z-score-normalized expression of top cluster-specific genes. Representative GO terms are shown for each major cluster: “carbohydrate catabolic process” (PT cells), “antigen processing and presentation” (macrophages), “leukocyte chemotaxis” (macrophages/neutrophils), “muscle contraction” (myofibroblasts), and “multicellular organismal homeostasis” (collecting duct cells).

To delineate the precise mechanism of TKT-mediated metabolic reprogramming in renal injury, we conducted a granular analysis at the single-cell level. Utilizing the public dataset GSE197266, which encompasses various classic male mouse AKI models—including IRI, CP, FA, SO, and UUO —we analyzed renal samples collected 48 h post-injury to capture peak transcriptomic features. Rigorous quality control yielded 77,593 high-quality single cells. Samples exhibited high statistical consistency in nFeature_RNA and nCount_RNA distributions, with mitochondrial gene fractions maintained below 6%, confirming superior cell viability and sequencing depth (Figure S1A). To mitigate batch effects across induction models, we utilized the Harmony algorithm for integration and performed dimensionality reduction via the top 30 principal components, achieving a seamless fusion of cell groups in two-dimensional space (Figure S1B–D and Figure 3A). Based on lineage-specific markers, 16 major cell sub-clusters were successfully identified. The UMAP plot clearly illustrates the heterogeneity of renal tissue, identifying populations such as Proximal Tubule Cells (PTCs; Slc34a1^+^/Lrp2^+^), Macrophages (C1qa^+^/Adgre1^+^), T cells (Ptprc^+^/Cd3d^+^), NK cells (Gzma^+^/Ncr1^+^), Podocytes (Nphs1^+^/Nphs2^+^), and Endothelial cells (Flt1^+^/Kdr^+^), among others (Figure 3B,C). Notably, an analysis of Tkt expression across these clusters (Figure S1E) revealed that Tkt is most prominently expressed in macrophages, showing both superior intensity and frequency compared to other populations. This specific distribution suggests that TKT-mediated carbohydrate metabolism may be a critical factor in macrophage-driven AKI inflammatory or repair responses. Pathway enrichment analysis confirmed the accuracy of our annotations, with sub-clusters significantly enriched in biologically relevant processes: PTCs in “carbohydrate catabolic processes,” macrophages in “antigen processing and presentation,” and collecting duct cells in “multicellular organismal homeostasis” (Figure 3D).

### 2.4. Macrophage Sub-Cluster Analysis and Dynamic Evolution of Tkt Expression During AKI

**Figure 4:**
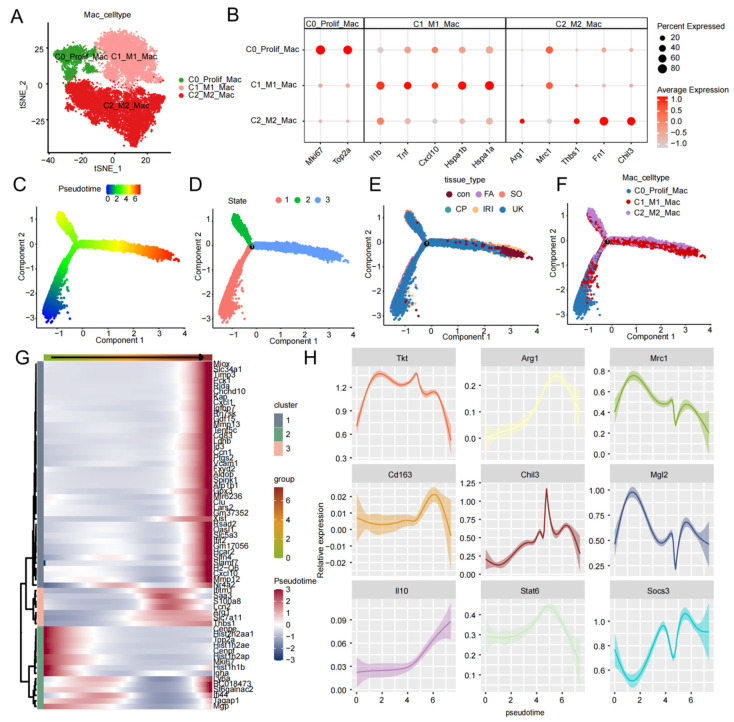
Subclustering and pseudotime trajectory analysis of macrophages across normal and AKI tissues. (**A**) t-SNE visualization of macrophage subclusters. Macrophages from both normal (Control) and AKI (FA, IRI, CP, SO, UUO) tissues were integrated and re-clustered into three functional subsets: C0_Prolif_Mac (proliferating macrophages), C1_M1_Mac (pro-inflammatory M1 macrophages), and C2_M2_Mac (anti-inflammatory/pro-repair M2 macrophages). (**B**) Dot plot of subtype-specific markers confirming the identity of each subset: Mki67 and Top2a for C0 (proliferating); *Il1b*, *Tnf*, and *Cxcl10* for C1 (M1); and *Arg1*, *Mrc1*, and *Fn1* for C2 (M2). (**C**–**F**) Monocle2 pseudotime trajectory of macrophage polarization. The trajectory illustrates the evolutionary continuum of macrophages from a normal physiological state toward a pathological and reparative state. Cells are colored by pseudotime score (**C**), inferred differentiation state (**D**), tissue origin (**E**), and cell subtype (**F**). (**G**) Pseudotime-dependent heatmap showing genes whose expression significantly shifts during the transition. Early-phase genes (*Mki67*, *Top2a*, *Ly6a*) are associated with proliferation and defense response; mid-phase genes (*S100a8*, *Lcn2*, *Arg1*) are involved in emergency response and early repair; late-phase genes are linked to mature repair and metabolic remodeling. (**H**) Dynamic expression profiles of hub and marker genes along the pseudotime axis. Tkt expression increases significantly during the transition and peaks alongside M2-specific markers (*Arg1*, *Mrc1*, *Mgl2*), suggesting a coordinated role in the M2 polarization process. The x-axis represents pseudotime progression, and the y-axis represents relative expression levels.

To precisely resolve the *Tkt*-mediated metabolic reprogramming of macrophages, we performed deep clustering and pseudotime analysis on the macrophage population. Utilizing t-SNE dimensionality reduction, the macrophages were categorized into three distinct functional subsets (Figure 4A): C0_Prolif_Mac (proliferative, expressing *Mki67* and *Top2a*), C1_M1_Mac (pro-inflammatory, high in *Il1b*, *Tnf*, and *Cxcl10*), and C2_M2_Mac (anti-inflammatory/repair, expressing *Arg1*, *Mrc1*, and *Fn1*) (Figure 4B). Analysis of *Tkt* distribution confirmed its highest expression and coverage within the C2_M2_Mac sub-cluster (Figure S1F). Monocle-based pseudotime trajectories (Figure 4C–F) revealed a clear branching evolution from early proliferative or inflammatory states toward a repair phenotype. To identify the key drivers of this transition, we generated a pseudotime-dependent heatmap (Figure 4G), which exhibited dynamic switching of gene clusters. In the early phase, genes were primarily concentrated in cell proliferation and defense response pathways (e.g., *Mki67*, *Top2a*, *Ly6a*). The middle phase was characterized by pulse-like expression of genes involved in emergency response and early repair, such as *S100a8*, *Lcn2*, and *Arg1*. In the late stage, a gene cluster associated with mature repair and metabolic remodeling was persistently upregulated. Dynamic expression analysis along the pseudotime axis (Figure 4H) showed that *Tkt* is rapidly induced at the trajectory onset and maintained at high levels within the repair branch. Crucially, the expression kinetics of *Tkt* were highly synchronized with repair markers such as *Arg1*, *Mrc1*, and *Mgl2*, with significantly higher relative expression in the mid-to-late stages compared to the baseline. This spatio-temporal pattern suggests that *Tkt* may potentially drive the M2 polarization of macrophages during the AKI repair phase, providing a transcriptomic basis for investigating DS-DH components targeting TKT.

### 2.5. Cell–Cell Communication Analysis of Macrophage Subsets Based on Tkt Expression

**Figure 5:**
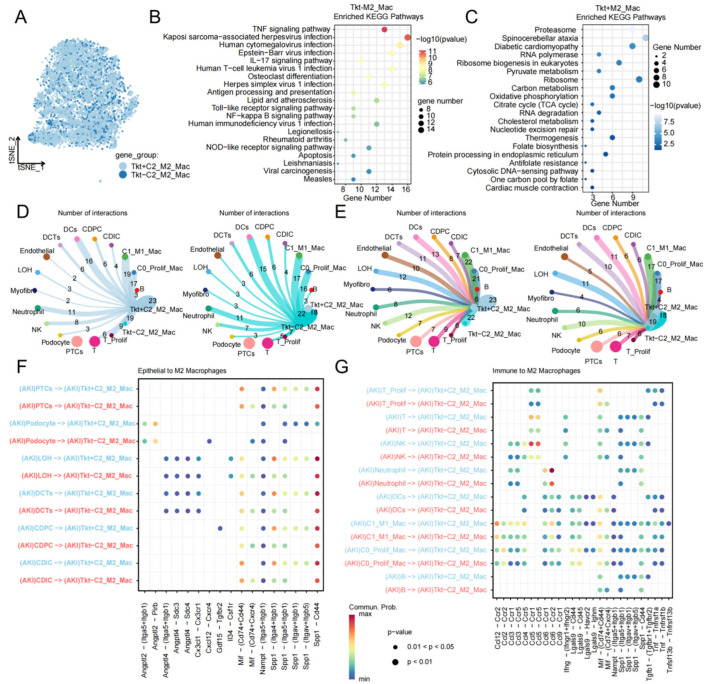
Cell–cell communication landscape and functional divergence of *Tkt*^+^ vs. *Tkt*^−^ M2 macrophages. (**A**) t-SNE visualization of M2 macrophage subsets stratified by *Tkt* expression. M2 macrophages were categorized into *Tkt*^+^ M2_Mac (high Tkt expression) and *Tkt*^−^ M2_Mac (low/absent *Tkt* expression) subclusters. (**B**,**C**) Distinct KEGG functional enrichment profiles of the two subsets. (**B**) *Tkt*^+^ M2 macrophages are enriched in carbon metabolism, the TCA cycle, and oxidative phosphorylation. (**C**) *Tkt*^−^ M2 macrophages are enriched in proteasome, pyruvate metabolism, oxidative phosphorylation, and apoptosis pathways. (**D**,**E**) Global interaction strength analysis visualized by chord diagrams. (**D**) Incoming and outgoing signaling patterns of *Tkt*^+^ M2_Mac cells. (**E**) Signaling patterns of Tkt^−^ M2_Mac cells, showing the superior connectivity of the *Tkt*^+^ population. (**F**,**G**) Ligand–receptor pair interaction analysis. (**F**) Interactions between renal epithelial cells (PTC, proximal tubule cells; LOH, loop of Henle; CDPC, collecting duct principal cells) and M2 subsets, showing that the *SPP1-CD44* and SPP1–integrin axes are predominantly active in *Tkt*^+^ M2_Mac cells. (**G**) Communication from immune cells to macrophages, demonstrating that *Tkt*^+^ M2_Mac cells possess higher sensitivity to CCL, MIF, and NAMPT signaling pathways. Edge thickness in chord diagrams corresponds to interaction strength.

To further decode the functional network of *Tkt* within the AKI repair microenvironment, the CellChat algorithm was employed to evaluate interactions between *Tkt*^+^ M2 macrophages (*Tkt*^+^ C2_M2_Mac) and other cell types. Pathway enrichment analysis indicated that *Tkt*^+^ C2_M2_Mac are significantly enriched in fundamental metabolic pathways, including carbon metabolism, the TCA cycle, and oxidative phosphorylation (Figure 5B), reflecting an active energetic state. Conversely, *Tkt*^−^ C2_M2_Mac were more closely associated with inflammatory regulation (TNF, IL-17, and Toll-like receptor signaling) and immune processes like antigen presentation (Figure 5C). Global communication network analysis showed that *Tkt*^+^ C2_M2_Mac possess extensive interaction potential, particularly with PTCs, endothelial cells, and T cells (Figure 5D,E). Ligand–receptor pair analysis further demonstrated that epithelial-derived molecules such as *Angpt2/4*, *Spp1*, and *Mif* interact strongly with their corresponding receptors on *Tkt*^+^ C2_M2_Mac (Figure 5F). Additionally, within the immune cell network (Figure 5G), *Tkt*^+^ C2_M2_Mac maintain tight homeostatic regulation with T cells and neutrophils via canonical signaling axes such as *Cd12*, *Ifng*, *Tnf*, and *Spp1-Cd44*. These findings suggest that high *Tkt* expression defines a unique metabolic state in M2 macrophages, enabling them to function as central communication hubs in the restoration of renal homeostasis following AKI.

### 2.6. Molecular Docking Validation of Active Compounds and TKT Protein

**Figure 6:**
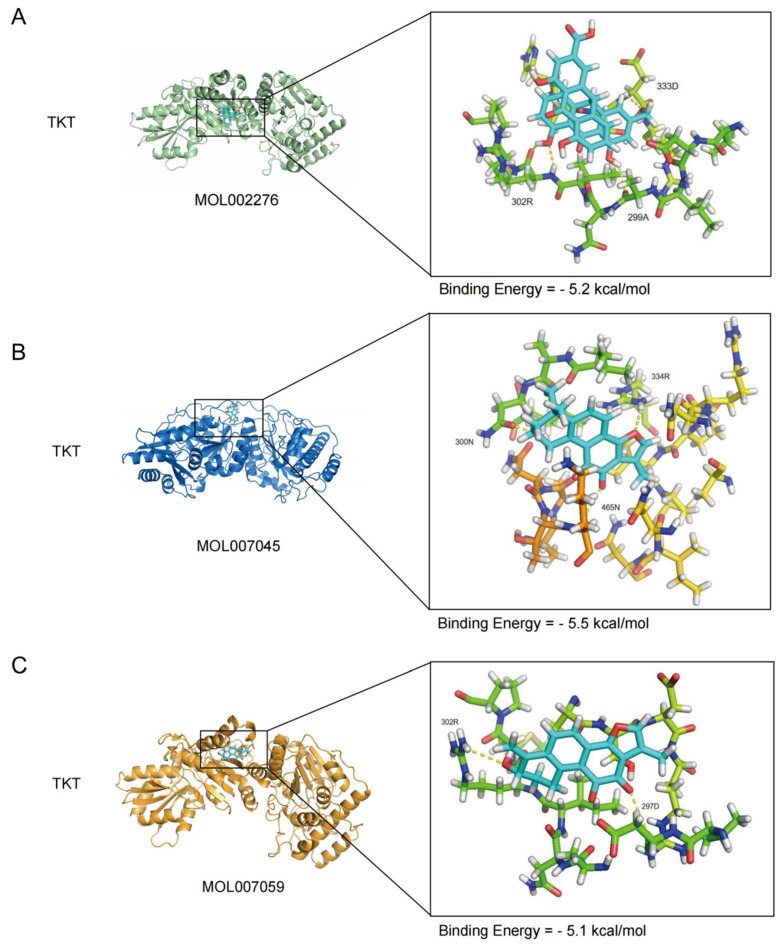
Molecular docking visualization of TKT with active compounds derived from DS-DH. The structural interactions between the hub protein TKT and representative active ingredients from DS-DH are presented, where the left panels display the overall surface or ribbon conformations of the protein–ligand complexes and the right panels provide magnified views of the binding pockets. Specifically, the binding orientations and critical amino acid residues are shown for the TKT–MOL002276 complex (**A**), the TKT–MOL007045 complex (**B**), and the TKT–MOL007059 complex (**C**). Dashed lines indicate the predicted hydrogen-bonding interactions within the active site, with corresponding binding energy values (kcal/mol) and key interacting residues labeled accordingly.

To validate the interaction potential between core active components of DS-DH and the TKT protein at the molecular level, we utilized molecular docking to assess the binding affinity of candidate compounds within the TKT active pocket. Results demonstrated that the primary active components—MOL002276, MOL007045, and MOL007059—precisely fit into the TKT binding site, exhibiting favorable molecular recognition (Table 1). Specifically, MOL007045 showed the highest binding stability with a minimum binding energy of −5.5 kcal/mol, forming strong hydrogen bonds and hydrophobic interactions with key residues 334R, 300N, and 465N within the TKT protein (Figure 6B). Furthermore, MOL002276 achieved a binding energy of −5.2 kcal/mol, interacting primarily with 302R, 299A, and 333D (Figure 6A), while MOL007059 recorded −5.1 kcal/mol, stabilizing its conformation via interactions with residues 302R and 297D (Figure 6C). These docking simulations confirm that the active constituents of DanShen and DaHuang possess the structural basis to directly target the TKT protein. Combined with our single-cell findings, this suggests that these components may regulate macrophage functional states by modulating TKT-mediated metabolic pathways.

## 3. Discussion

AKI is a complex syndrome characterized by rapid renal functional decline and severe tissue damage. We recently demonstrated in a cisplatin-induced murine model that the DS-DH herb pair effectively attenuates AKI by modulating the gut–kidney axis and broadly inhibiting the MAPK signaling pathway [32]. While that study provided a macro-level pharmacological landscape, the present work was designed to address the subsequent critical question: at the single-cell level, what are the specific cellular targets within the renal microenvironment that initiate these therapeutic effects? To answer this, we employed an integrative computational framework combining network pharmacology, multi-algorithm machine learning, and single-cell transcriptomics to systematically predict and prioritize potential molecular targets of the DS-DH herb pair. Through this pipeline, TKT emerged as the top-ranked feature gene from 62 initial candidates, and scRNA-seq analysis revealed its predominant enrichment in M2-polarized renal macrophages.

The identification of TKT as a highly ranked feature via an ensemble of machine learning algorithms (including LASSO, Boruta, and Random Forest) suggests its close association with AKI pathology. TKT is a key enzyme in the non-oxidative branch of the pentose phosphate pathway (PPP), participating in the generation of ribose-5-phosphate for nucleotide synthesis and the maintenance of cellular redox balance [33]. While primarily investigated in cancer metabolic reprogramming [34], its regulatory patterns in acute inflammatory conditions, such as AKI, remain to be fully characterized. Our cross-algorithm analysis highlighted TKT as a prominent feature within the disease state, indicating that metabolic alterations, specifically within carbohydrate catabolism, might serve as relevant interventional nodes for the DS-DH formulations. Notably, the PPP is a key source of NADPH for cellular antioxidant defense, providing a potential mechanistic link to our previous finding that DS-DH attenuated oxidative stress in the AKI model [32].

At the single-cell level, transcriptomic mapping demonstrated that *Tkt* expression is notably enriched within the macrophage population. Macrophages are pivotal in the AKI microenvironment, exhibiting phenotypic plasticity as they transition from a pro-inflammatory (M1) to a reparative (M2) state [35]. This transition is frequently accompanied by metabolic rewiring, where M2 macrophages tend to rely on oxidative phosphorylation and the PPP to sustain the energetic and biosynthetic demands of tissue repair [36,37]. Building on this metabolic framework, single-cell analysis further showed that *Tkt* was most highly enriched in the M2 macrophages and increased progressively along the reparative trajectory in parallel with *Arg1*, *Mrc1*, and *Mgl2*. This spatiotemporal pattern suggests that *Tkt* may be intimately associated with the emergence of the M2 reparative program and could potentially serve as a metabolic facilitator of this phenotypic transition. However, it is important to emphasize that the current data demonstrate correlation rather than causation; whether TKT actively drives M2 polarization or is simply a consequence of the M2 metabolic state requires future investigation using macrophage-specific *Tkt* knockout models. With this caveat in mind, it is worth noting that, should TKT modulation be experimentally confirmed, the anti-inflammatory and antioxidant effects of DS-DH constituents could be partly explained by the TKT-driven M2 macrophage polarization and pentose phosphate pathway flux, which supplies NADPH to sustain cellular redox defense. The synchronized upregulation of *Tkt* with *Arg1* and *Mrc1* along the reparative trajectory further suggests that DS-DH may reinforce endogenous repair and attenuate the AKI-to-CKD transition, a hypothesis consistent with the *PI3K-Akt* and leukocyte chemotaxis pathways enriched in our network pharmacology results.

Furthermore, we explored the intercellular communication networks within the reparative niche. In AKI, crosstalk between proximal tubule cells (PTCs) and macrophages is increasingly recognized as an important component of the repair microenvironment. Previous studies have shown that MIF- and SPP1-related signaling are major communication routes from injured or proliferating proximal tubule cells to myeloid cells, highlighting the active role of tubular epithelial cells in shaping macrophage behavior during renal repair [38]. CellChat analysis revealed predicted interactions between *Tkt*^+^ M2 macrophages and other renal lineages, such as PTCs, through signaling axes like *Spp1* and *Mif* [30]. These transcriptomic phenomena suggest that *Tkt*^+^ macrophages might participate as active communication nodes in the epithelial-immune dialogue during renal recovery [39,40]. In other words, high *Tkt* expression in M2-like macrophages may not only reflect an intracellular metabolic state but also indicate their responsiveness to epithelial-derived repair cues and their role in coordinating renal recovery. However, it is important to note that these interactions are computationally inferred and represent observed phenomena at the mRNA level, necessitating future in vitro and in vivo functional assays to validate these paracrine networks.

To evaluate the feasibility of DS-DH components targeting TKT, we performed molecular docking simulations. The core active constituents from DanShen and DaHuang (e.g., MOL007045, MOL002276) exhibited favorable binding affinities to the active pocket of the TKT protein [41]. This provides a theoretical structural basis for potential interactions between these herbal compounds and TKT. Given the central role of TKT in the non-oxidative branch of the pentose phosphate pathway, these findings raise the possibility that DS-DH may influence macrophage-associated metabolic remodeling through TKT. While these predictive models suggest a possibility that DS-DH might influence the PPP flux in macrophages, these findings remain strictly predictive and require comprehensive experimental confirmation.

Several limitations in the current study must be explicitly acknowledged. First, the core targets, developmental trajectories, and cell–cell communication networks were derived entirely from computational predictions and public transcriptomic datasets without independent biological validation [42]. The findings should therefore be interpreted as a high-confidence guide for future research, not as definitive proof of a mechanism. Second, the observed correlation between *Tkt* expression and M2 macrophage polarization does not establish a causal relationship; the most critical next step is in vivo validation using macrophage-specific *Tkt* conditional knockout mice to determine whether TKT is indispensable for the nephroprotective efficacy of DS-DH during AKI. Third, while molecular docking indicates binding potential, the precise thermodynamic binding kinetics and the actual modulatory effects of these compounds on TKT enzymatic activity require future empirical validation through techniques such as surface plasmon resonance (SPR) or enzymatic activity assays. Finally, the mechanistic link between TKT-mediated metabolic reprogramming and the previously identified MAPK pathway inhibition remains hypothetical, warranting dedicated biochemical experiments to elucidate their potential crosstalk.

## 4. Materials and Methods

### 4.1. Data Acquisition

In this study, two transcriptomic datasets were retrieved from the Gene Expression Omnibus (GEO) database: a scRNA-seq dataset (GSE197266), which encompasses various mouse models of AKI, and a bulk transcriptomic dataset (GSE30718), consisting of 28 AKI samples and 8 healthy controls.

### 4.2. Screening of Bioactive Components and Target Prediction

The active constituents of DanShen and DaHuang were sourced from the Traditional Chinese Medicine Systems Pharmacology (TCMSP) database. Candidates were filtered based on strict criteria: oral bioavailability (OB) ≥ 30% and drug-likeness (DL) ≥ 0.18, resulting in 65 compounds from DanShen and 17 from DaHuang. Potential pharmacological targets for these 82 compounds were predicted using the SwissTargetPrediction database, identifying 1058 candidate targets. Concurrently, AKI-related genes were obtained from the GeneCards database using the keyword “Acute Kidney Injury”, with a threshold relevance score > 10.

### 4.3. Weighted Gene Co-Expression Network Analysis (WGCNA)

Weighted Gene Co-expression Network Analysis (WGCNA) [43] was employed to identify key gene modules associated with AKI. After quality control of the bulk transcriptomic data, an optimal soft-thresholding power was determined to construct a weighted adjacency matrix. Subsequently, hierarchical clustering was performed based on the Topological Overlap Matrix (TOM), and co-expression modules were identified using the dynamic tree cut method (minimum size = 500). Finally, Module Eigengenes (MEs) were correlated with clinical traits to identify core modules significant to AKI pathology.

### 4.4. Single-Cell RNA-Seq Data Pre-Processing

The scRNA-seq data were systematically processed using the Seurat (v4.2.0) [44] R package. Initially, rigorous quality control was implemented to exclude low-quality cells, retaining only those with 500–4000 detected genes (nFeature_RNA) and a mitochondrial gene content (percent.mt) below 6%. To ensure data accuracy, potential doublets were identified and removed using the DoubletFinder package. Following quality control, the data were normalized via the LogNormalize method, and the top 2000 highly variable genes were identified and scaled for downstream analysis.

Dimensionality reduction was performed using Principal Component Analysis (PCA) with the top 30 principal components, and the Harmony algorithm was employed to correct for batch effects across different samples. Based on the integrated feature space, cell clustering was conducted using FindNeighbors and FindClusters with a resolution of 0.8, followed by visualization via the UMAP algorithm. Finally, cell lineages were identified through manual annotation based on the expression of canonical marker genes.

### 4.5. Cell–Cell Communication Analysis

To systematically characterize interpersonal cellular communication patterns, we employed the CellChat R package (v1.6.1) [45], which facilitates the inference and quantitative analysis of intercellular communication networks from scRNA-seq data.

### 4.6. Pseudotime Trajectory Analysis

The Monocle2 R package (v2.28.0) [46] was utilized to perform trajectory analysis, revealing the developmental lineages and dynamic state transitions of specific cell populations during AKI progression.

### 4.7. Functional Enrichment Analysis

Gene Ontology (GO) and Kyoto Encyclopedia of Genes and Genomes (KEGG) pathway analyses were performed to predict the biological functions and signaling pathways of target genes. These analyses were implemented using the clusterProfiler R package [47]. Enrichment significance was determined by a threshold of *p* < 0.05 and a False Discovery Rate (FDR, q-value) < 0.25.

### 4.8. Molecular Docking Validation

To elucidate the interaction modes between core constituents of the DS-DH pair and their corresponding target proteins, molecular docking simulations were conducted. Representative active compounds were selected as ligands based on their topological significance within the component–target network. High-resolution 3D protein structures were retrieved from the Protein Data Bank (PDB), and chemical structures of the ligands were sourced from PubChem in SDF format. Proteins underwent standardized pre-processing, including dehydration, hydrogenation, and charge balancing. Semi-flexible docking was executed via AutoDock Vina software 1.2.6, with binding affinities quantified by the minimum binding energy. Optimal docking poses were visualized using PyMOL 2.6.2 to characterize hydrogen bonding networks and hydrophobic interactions at the active site.

### 4.9. Statistical Analysis

GraphPad Prism 8.0 (GraphPad Software, San Diego, CA, USA) and R software v4.3.1 (R Foundation for Statistical Computing, Vienna, Austria) were used for statistical analyses. A *p*-value < 0.05 was considered statistically significant (* *p* < 0.05, ** *p* < 0.01).

## 5. Conclusions

Our results showed that the DS-DH herb pair might mitigate AKI by potentially targeting TKT, which appears to be closely associated with the metabolic reprogramming and M2 polarization of macrophages. These findings offer a preliminary immunometabolic perspective on the synergistic mechanisms of TCM and suggest TKT as a candidate target for future experimental exploration in acute renal injury.

## Figures and Tables

**Table 1:** Binding energies of major active components from DanShen and DaHuang with TKT protein.

MOL_Name	Drug	Molecule Name	Binding Energy (kcal/mol)
MOL002276	Dahuang	Sennoside E_qt	−5.2
MOL007045	Danshen	3α-hydroxytanshinoneIIa	−5.5
MOL007059	Danshen	3-beta-Hydroxymethyllenetanshiquinone	−5.1

## Data Availability

All data processing and analysis codes are available from the corresponding author upon reasonable request.

## Supplementary Materials

The following supporting information can be downloaded at https://www.mdpi.com/article/10.3390/ijms27104435/s1.
