# Supplementary material for: Integrated Network Pharmacology and Single-Cell Transcriptomics Reveal Transketolase as a Potential Target for the DanShen–DaHuang Herb Pair in Acute Kidney Injury
Source: Int J Mol Sci. 2026 May 15;27(10):4435. doi: 10.3390/ijms27104435 (PMC13207515; doi:10.3390/ijms27104435)
Supplement: Supplementary file 1 [file ijms-27-04435-s001.zip › ijms-4269310 supplementary figure.pdf]

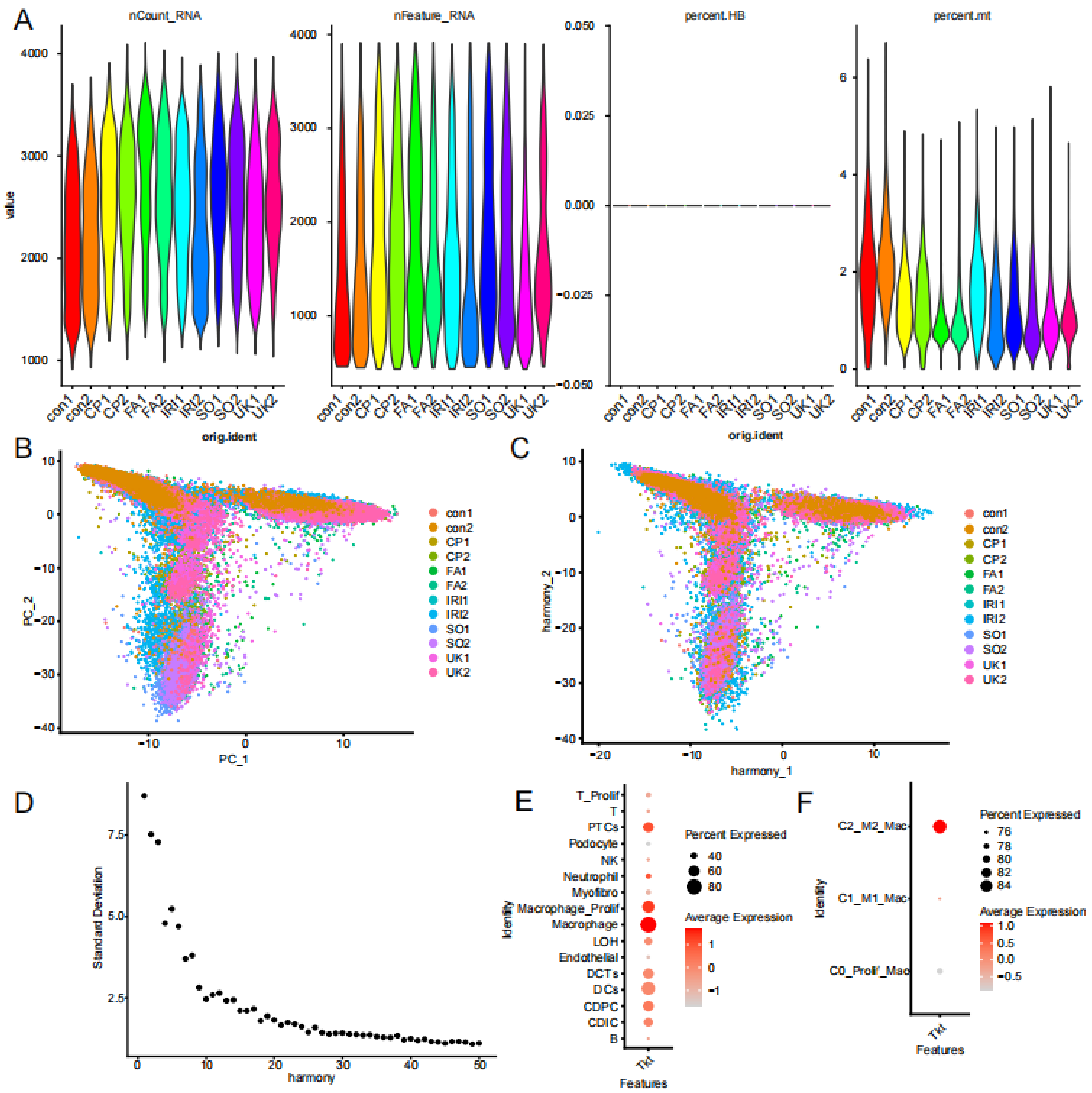

Figure S1. Quality control and integration analysis of scRNA-seq. (A) 1. CP1-2, FA1-2, IRI1-2, SO1-2, UK1-2) after quality control; (B-C) Dimensional reduction plots of cells before (B, based on PCA) and after (C) integration using the Harmony algorithm, colored by sample origin; (D) Elbow plot showing the standard deviation of the first 50 principal components (PCs) following Harmony integration; (E-F) Dot plots illustrating the expression profiles of Tkt across 16 major kidney cell types (E) and three macrophage subsets (F), where dot size represents the percentage of expressing cells and color intensity represents the average expression level.
